# Alteration of mechanical stresses in the murine brain by age and hemorrhagic stroke

**DOI:** 10.1093/pnasnexus/pgae141

**Published:** 2024-04-24

**Authors:** Siyi Zheng, Rohin Banerji, Rob LeBourdais, Sue Zhang, Eric DuBois, Timothy O’Shea, Hadi T Nia

**Affiliations:** Department of Biomedical Engineering, Boston University, Boston, MA, USA; Department of Biomedical Engineering, Boston University, Boston, MA, USA; Department of Biomedical Engineering, Boston University, Boston, MA, USA; Department of Biomedical Engineering, Boston University, Boston, MA, USA; Department of Biomedical Engineering, Boston University, Boston, MA, USA; Department of Biomedical Engineering, Boston University, Boston, MA, USA; Department of Biomedical Engineering, Boston University, Boston, MA, USA

**Keywords:** residual solid stress, mouse brain, age-related alteration, hemorrhagic stroke

## Abstract

Residual mechanical stresses, also known as solid stresses, emerge during rapid differential growth or remodeling of tissues, as observed in morphogenesis and tumor growth. While residual stresses typically dissipate in most healthy adult organs, as the growth rate decreases, high residual stresses have been reported in mature, healthy brains. However, the origins and consequences of residual mechanical stresses in the brain across health, aging, and disease remain poorly understood. Here, we utilized and validated a previously developed method to map residual mechanical stresses in the brains of mice across three age groups: 5–7 days, 8–12 weeks, and 22 months. We found that residual solid stress rapidly increases from 5–7 days to 8–12 weeks and remains high in mature 22 months mice brains. Three-dimensional mapping revealed unevenly distributed residual stresses from the anterior to posterior coronal brain sections. Since the brain is rich in negatively charged hyaluronic acid, we evaluated the contribution of charged extracellular matrix (ECM) constituents in maintaining solid stress levels. We found that lower ionic strength leads to elevated solid stresses, consistent with its unshielding effect and the subsequent expansion of charged ECM components. Lastly, we demonstrated that hemorrhagic stroke, accompanied by loss of cellular density, resulted in decreased residual stress in the murine brain. Our findings contribute to a better understanding of spatiotemporal alterations of residual solid stresses in healthy and diseased brains, a crucial step toward uncovering the biological and immunological consequences of this understudied mechanical phenotype in the brain.

**Significance Statement**

While emerging evidence highlights the importance of solid stresses in embryogenesis and tumor growth, the genesis and consequences of residual solid stresses in the adult normal brain remain poorly understood. Understanding the spatiotemporal distribution and alteration of the residual solid stresses as the brain ages and is impacted by neuropathologies, such as a stroke, will elucidate the biological and immunological consequences of maintaining these stresses. This study suggests solid stress could serve as a potential biomarker in aging and diseases associated with the brain.

## Introduction

Residual solid stresses are the compressive and tensile mechanical stresses generated and transmitted by solid components of tissue as opposed to interstitial fluid pressure (1, 2). Differential growth, cell–cell and cell–matrix interactions, and electroosmotic stresses involved in organ development and disease progression give rise to residual mechanical stresses (3–7). Residual solid stresses in biological tissues were first identified in arteries, where changes in systemic pressure can trigger asymmetrical growth in the inner and outer layers (8), and later also found during rapid growth, a defining feature of organogenesis (5, 7–10) and tumor growth (1, 3, 11, 12). During tumor growth, residual solid stresses were shown to be a key contributor in tumor progression (13, 14), immune evasion (15), and treatment resistance by directly compressing the blood and lymphatic vessels (12, 16, 17) or directly compressing the cancer cells (18, 19). More recently, we showed that solid stresses applied on the neurons surrounding brain tumors causes neuronal death (20, 21), which can be protected by neuroprotective strategies such as the use of lithium (20). While elasticity, a traditionally studied biomechanical property, has been extensively probed in brain development and pathogenesis (22–25), our understanding of the genesis and consequences of residual solid stress is limited in brain.

When the growth and remodeling of tissues are slowed down, as in most adult organs, solid stresses appear to dissipate as previously shown in liver and kidneys (3); in contrast, solid stresses in the adult brain are maintained at high levels. The initial evidence on the existence of solid stresses in the adult brain was shown by the partial cut method (26). In a later work, it was shown that the differential growth in brains, synthetically modeled by polymer expansion, is responsible for the brain folding (27). Additionally, mechanical tension in the brain is shown to be an important signal for neuromuscular synapses to modulate vesicle accumulation and synaptic plasticity (28). In a recent study where the temporal volumetric change of different brain anatomical compartments was quantified over the lifespan of over 100,000 humans (29), differential volumetric changes of key components of the brain were reported: (i) gray matter reached its maximum volume in childhood (1–3 years), while white matter reached its maximum volume in young adulthood (20–30 years) and (ii) while the gray matter, white matter, and cortical volumes decline in late adulthood (60–100 years), the ventricle volume dramatically increases during this time. The differential volumetric changes imply the existence of residual solid stresses in the human brain. The brain also undergoes substantial morphological and structural alterations in diseases such as Alzheimer's (24, 30, 31), stroke (32–34), scar formation (22), and cancer (20, 21) that potentially give rise to residual mechanical stresses. Key unanswered questions are how the residual solid stress changes in these diseases, and whether these mechanical stresses contribute to the progression of the disease. Our limited understanding of the solid stress-associated mechanobiology in the brain is partially due to challenges in directly mapping these stresses. Unlike stiffness, a mechanical property which has been successfully demonstrated as a key player in the brain health and disease (22–25), measuring the solid stress requires either mechanical intervention to relax the existing stresses (3, 20, 35) or embedding microgels to estimate the stresses (18, 36–39).

Here, we use our previously developed method (3, 35) to map the solid stresses in a mouse brain at three different time points during its lifespan: early childhood (5–7 days), adulthood (8–12 weeks), and late adulthood (22 months). Our method, based on relaxing solid stresses by slicing the freshly resected brain into thin sections, provides the deformation induced by stress relaxation, which is quantified as (i) the normalized deformation, (ii) average local curvature, and (iii) changes in surface area. We then studied viscoelastic properties of the brain at these age groups through an unconfined compression test to obtain Young's modulus, rate-dependent stiffening, and stress relaxation time constant of the brain and their alteration by age. To better understand the determinants of solid stresses in brain, we then investigated the effect of ionic strength on the residual solid stress in the mouse brain and found that lower ionic strength results in higher level of solid stress, suggesting that charged extracellular matrix (ECM) constituents may contribute to the maintenance of solid stress in brain. We evaluated the 3D distribution of the residual solid stress in a series of coronal slices along the sagittal plane and found that solid stresses increase from anterior to posterior sections. Finally, we studied brains with a hemorrhagic stroke and found that regions with stroke have lower levels of residual solid stress. The unique approach and conceptual findings of this study will contribute to a better understanding of the origin and biological consequence of solid stress, a potential biomarker in brain aging and pathologies.

## Results

### Residual solid stress in the murine brain increased with age

**Fig. 1.**
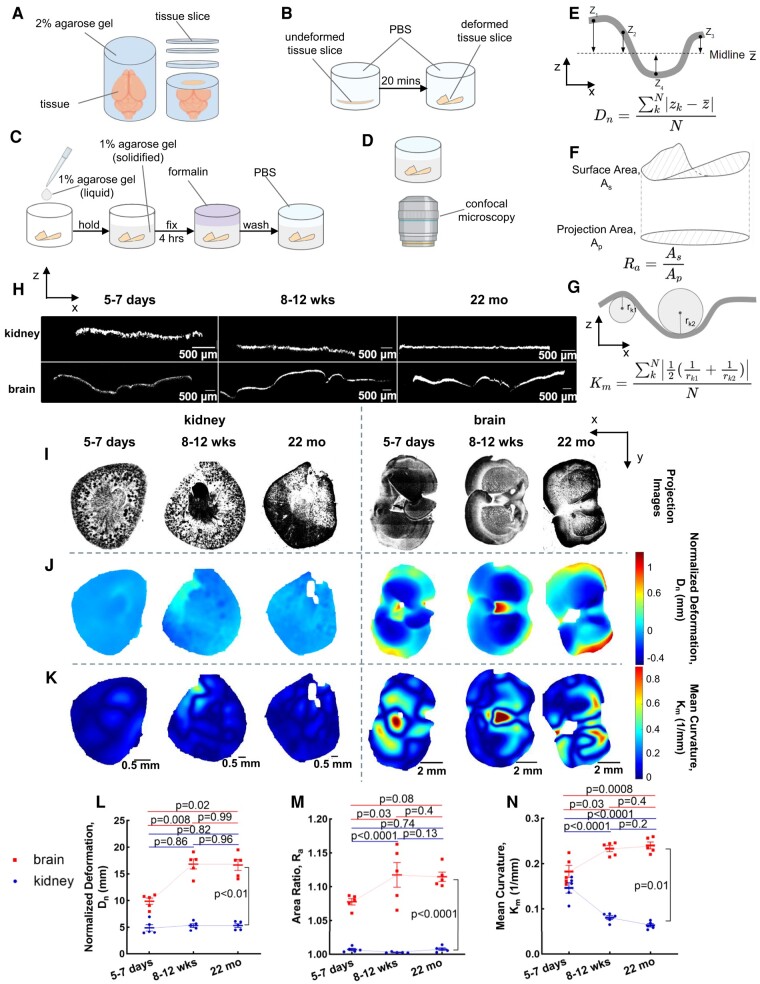
Residual solid stresses in murine brain are age-dependent. A) The fresh brain (graphic generated using BioRender.com) is embedded in 2% agarose, then sliced with a Compresstome to obtain a specific thickness of 250 μm. B) Slices are left in a PBS at room temperature for 20 min to deform as the solid stress is released in the tissue slice. C) The deformed slices are embedded in 1% agarose, then fixed with formalin overnight, and washed with PBS. D) The deformed slice is imaged via confocal microscopy. E) The normalized deformation, *D*_n_, an index of residual solid stress, is defined as the average height difference from curved surface to the midline. F) The area ratio, *R*_a_, defined as the ratio of surface area and projection area, is used as another index of solid stress. G) The curvature, *k*, defined as the reciprocal of the curvature radius, is used as the third index of solid stress. Mean curvature, *K*_m_, is the average of mean curvature on tissue slices. H) The orthogonal views of deformation of representative brain and kidney (negative control) slices from 5–7 days, 8–12 weeks, and 22 months mice. I) The projection images, J) deformation maps, and K) mean curvature maps of representative brain and kidney slices from 5–7 days, 8–12 weeks, and 22 months mice. L) Residual solid stresses are estimated in multiple ways by quantifying the normalized deformation, M) area ratio, and N) mean curvature. Brain slices have significantly higher normalized deformation, area ratio, and mean curvature than kidney slices in all age groups. Brain slices have increased normalized deformation, area ratio, and mean curvature from 5–7 days to 8–12weeks, and then keep plateau from 8–12 weeks to 22 months (mean ± SEM, *n* = 5 mice, two-tailed t test).

Based on the first principle that tissues containing residual solid stress deform upon release of physical confinement (3, 35), we sliced brains taken from mice ranging from 5–7 days to 22 months old to quantify the alteration of the residual solid stress as the brain matures. The harvested brain was embedded in agarose and then sliced into 250 µm thick sections that were left in phosphate-buffered saline (PBS) and separated from the surrounding agarose to allow the residual solid stresses to relax (Fig. 1A and B). The deformed tissue slice, after stress relaxation, was embedded in agarose and fixed with formalin to preserve the deformed structure for 3D fluorescent microscopy (Fig. 1C and D). The assessment of structural changes in the tissue involved quantifying (i) normalized deformation (Fig. 1E), (ii) changes in surface area (Fig. 1F), and (iii) local curvature (Fig. 1G). High-resolution fluorescent microscopy was employed to capture these changes comprehensively in 3D. The deformation or curvature maps are then projected onto a 2D plane as shown in Fig. 1J and K. The normalized deformation (Fig. 1E) served as a quantitative measure of solid stress, indicates the extent of bending in the tissue following slicing and stress relaxation. Comparing the total surface area of the deformed slice to its projected 2D area provided another measure of surface deformation (Fig. 1F). Finally, the quantification of local curvature on the 3D surface poststress relaxation (Fig. 1F) highlights the spatial heterogeneity of the solid stresses.

To validate our measurement method and rule out the effect of buoyancy in the measurement, we quantified and compared the solid stress indices, including normalized deformation, area ratio, and mean curvature, before and after flipping the slice and found no significant difference between the groups across all indices (Fig. S1). These results demonstrated that the buoyancy had no significant effect on deformation and solid stress quantification, and thus did not cause any artifact in our analysis. Next, we compared the solid stress indices in freshly excised vs. fixed slices of the same tissue and found no significant difference in the deformation and solid stress quantification (Fig. S2).

We compared the deformation of the brain slices against kidney (known to have negligible residual solid stress (3)) slices which remained flat with no local bending (Fig. 1H) taken from the same mouse. Representative deformation map of kidney slices from microscopy in each age group (Fig. 1I) showed a relatively flat surface with negligible deformation and curvature that was homogenously distributed across the tissue slice indicating lower residual solid stresses (Fig. 1J). Compared to this, there was substantially larger deformation (Fig. 1J) and curvature (Fig. 1K) that was heterogeneously distributed across the brain slices indicating higher residual solid stresses. The normalized deformation and area ratios across each age group for all experimental repeats was significantly higher for brain slices than the kidney as the negative control (Fig. 1L and M). The mean curvature, and the brain slices also had significant higher curvature than kidney slices (Fig. 1N) other than when the tissue was taken from 5–7 days old mice. Overall, the normalized deformation, area ratio, and mean curvature corroborate that the brain had a larger residual solid stress than the kidney. We also studied the heart as a positive control, as it is known to have high residual solid stress (10), and found that the heart slices showed greater and unevenly distributed deformation compared to kidney (Fig. S3).

Next, we evaluated the changes in the residual solid stress in brain with age. We found that the normalized deformation in brain slices increased significantly between the 5–7 days and 8–12 weeks age groups, and then remained constant between the 8–12 weeks and 22 months age groups (Fig. 1L). We observed consistent trend in other solid stress indices, the area ratio, and mean curvature (Fig. 1M and N). We expected the fast prenatal growth to give rise to higher growth-induced stresses, which would dissipate in slower postnatal brain development. We were surprised to find that the peak stress in the brain occurs long after birth up till late adulthood (22 months). Unlike other organs (e.g. kidney and liver) (3), the solid stress in healthy adult brains does not dissipate over time, and high levels of stress are maintained even into late adulthood. While we are aware of the major differences between the human and mouse brain, the differential change of volume of different brain components in humans (Fig. S4) can be informative in understanding the age-related dynamics of solid stress. The mechanical and biological characteristics of the brain change by age. Notably, there is a substantial increase in brain size from infancy to adulthood, encompassing both gray matter and white matter volumes (Fig. S4). Previous investigations have discerned differential growth patterns in gray and white matter during murine development, revealing tension in white matter and compression in gray matter, culminating in the generation of significant residual solid stress (26). Specifically, white matter volume reaches its peak at adolescence, and plateau during adulthood (40), which is similar to our findings that the residual solid stress increases during development and remains high through adulthood. Thus, the increasing white matter volume could be a potential determinant of residual solid stresses. Concurrently, research indicates that the mouse brain initiates neurogenesis around embryonic day 12, accompanied by the accumulation of hyaluronan around neurons, a phenomenon significantly diminished in adulthood (41). This alteration in hyaluronan levels might also influence the aggregation of solid stresses in the brain.

### Age-dependence of viscoelastic properties in murine brain

**Fig. 2.**
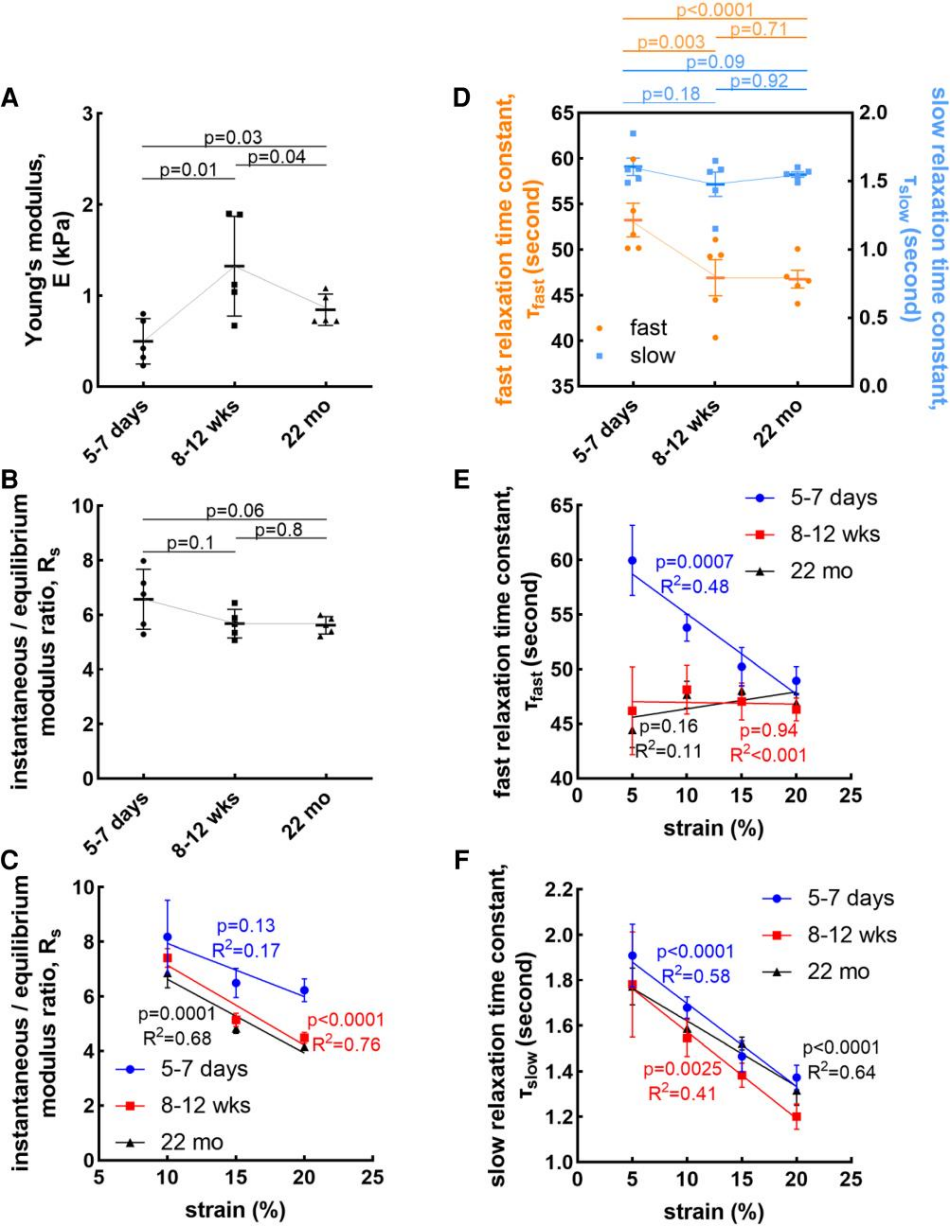
Viscoelastic properties in murine brain are age-dependent. A) Young's modulus and B) rate-dependent stiffening defined as instantaneous/equilibrium modulus ratio are age-dependent in brain (mean ± SEM, *n* = 5 mice, two-tailed t test). Brain from 8–12 weeks mice has the highest Young's modulus among all age groups. Instantaneous/equilibrium modulus ratio has a decreasing tendency in mice brains from 5–7 days to 22 months. C) The instantaneous/equilibrium modulus ratio is strain dependent (mean ± SEM, *n* = 5 mice, one-way ANOVA test). Brain tissue from 8–12 weeks and 22 months mice have decreasing tendency of instantaneous/equilibrium modulus ratio with strain. D) Fast relaxation time constant, and slow relaxation time constant, changes with age (mean ± SEM, *n* = 5 mice, two-tailed t test). Brain from 5–7 days mice has both the highest fast and slow relaxation time constants. The changing tendency of E) fast and F) slow relaxation time constant with strain (mean ± SEM, *n* = 5 mice, one-way ANOVA test).

Next, we asked whether the age-dependent changes in solid stress are associated with age-dependent alteration of material properties of the brain. We quantified the equilibrium and instantaneous Young's modulus, rate-dependent stiffening, and stress relaxation time constants of these properties in normal brain in the same age groups (Fig. S5). Unlike solid stress indices, equilibrium Young’s modulus significantly increased between the 5–7 days and 8–12 weeks age groups, and then decreased between the 8–12 weeks and 22 months age groups (Fig. 2A). The increase in Young's modulus between the 5–7 days and 8–12 weeks age groups was similar to a previous study that found human adult brain tissue was stiffer than those of children (42). Another study showed apparent elastic modulus increased with age in the mouse brain due to the increasing protein, lipid, and sulfated glycosaminoglycan content, whereas the water content decreased with age and negatively correlated with stiffness (43). While the increasing stiffness in this period is consistent with an increase in solid stress, the reduction of stiffness in the brain that happens in 22 months mice trends opposite to the elevated solid stress in that age group. Previous studies have established that white matter exhibits a softer consistency compared to gray matter within the brain (22, 44) which may provide a plausible correlation between the decline in gray matter volume (Fig. S4), and the reduction in overall brain stiffness as mice age from 8–12 weeks to 22 months.

The rate-dependent stiffening, measured as the ratio of instantaneous to equilibrium modulus and an indicator of brain viscoelasticity, showed only a decreasing trend (*P* = 0.06) from 5–7 days mice to 22 months mice (Fig. 2B). We reported the stress relaxation time constant in terms of fast and slow time constants, representing the early and late parts of the stress relaxation curves. The fast relaxation time constant decreased significantly from the 5–7 days to 8–12 weeks age groups, and remained low for the 22 months age group, while the slow relaxation time constant remained unchanged across age groups (Fig. 2D). This showed that mice in early infancy have longer time-dependent viscoelastic behavior compared to young and late adulthood. While the stress relaxation curve cannot be used to fully decouple intrinsic viscoelasticity (flow-independent) from poroelastic response (flow-dependent), the fast relaxation time constant is often associated to poroelastic behavior (45), which implies that the brain in early infancy has lower hydraulic permeability compared to later in life.

Since the relaxation of solid stresses results in large deformations (Fig. 1), we next probed whether the mechanical properties of the brain are linear or strain dependent. We observed that the rate-dependent stiffening and the relaxation time constants are all strain dependent in all the age groups (Fig. 2C, E, and F) except the fast relaxation time constant seen in the 8–12 weeks and 22 months groups. While the data demonstrate the highly nonlinear material properties of the brain across age groups, the underlying mechanisms need further investigation of the brain constituents and their response at high strains. Interestingly however, this nonlinear behavior remains mostly unchanged from infancy to late adulthood (Fig. 2C, E, and F).

### Residual solid stresses are higher in anterior than posterior sections of the murine brain

**Fig. 3.**
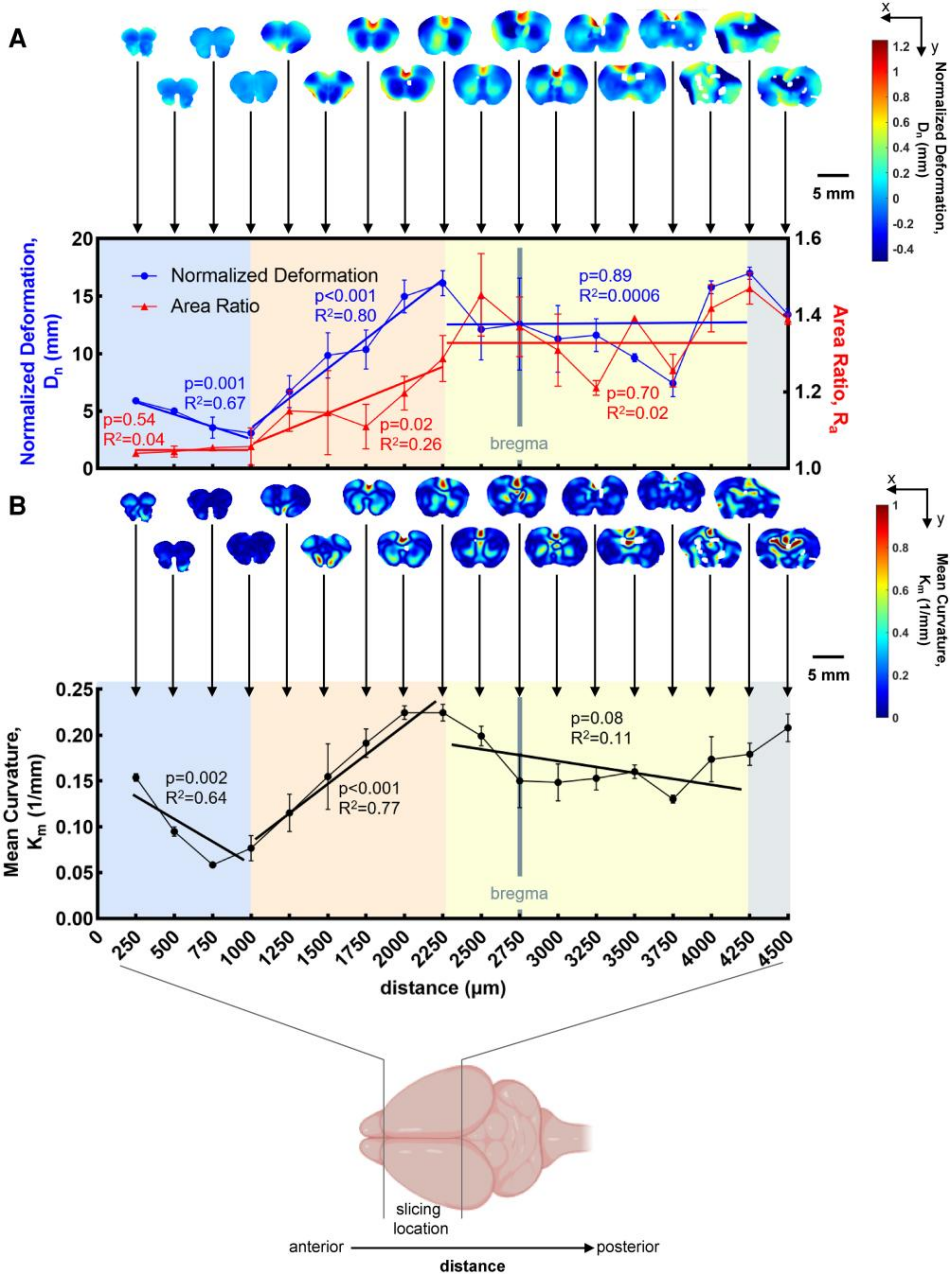
Residual solid stress distribution in the whole brain. A) The normalized deformation and area ratio of brain slices in the coronal direction (mean ± SEM, *n* = 3 mice, one-way ANOVA test) demonstrated a heterogenous distribution along the anterior–posterior axis. B) The mean curvature of brain slices in the coronal direction (mean ± SEM, *n* = 3 mice, one-way ANOVA test) demonstrated a heterogenous distribution along the anterior–posterior axis.

To better understand the spatial variation of the solid stress in brain, we probed the variation of solid stress from the anterior to posterior of the brain in 8–12 weeks old mice. We compared our results with previously published brain atlases that also used similar serial coronal sectioning (46–49). We categorized tissue slices into three different regions based on the changes in the dominant brain component, specifically 0–1,000 μm, 1,000–2,250 μm, and 2,250–4,250 μm, with the bregma situated at approximately 2,750 μm (Fig. 3).

In the 0–1,000 μm region, we observed a decrease in residual solid stress, including normalized deformation (Fig. 3A) and mean curvature (Fig. 3B). This region is predominantly composed of the isocortex and olfactory parts of the brain. The cross-sectional area of the isocortex trended to increase along the anterior–posterior axis, while the area of the olfactory region remained constant (Fig. S6). However, the residual solid stress increased with a significant positive slope in the 1,000–2,250 μm region across the normalized deformation, area ratio (Fig. 3A), and mean curvature (Fig. 3B). The deformation maps (Fig. 3A) and curvature maps (Fig. 3B) progressively showed greater heterogeneity in stress distribution in this region. Compared to the prior region, the cross-sectional area of the isocortex remained relatively constant, while the area of the striatum significantly increased (Fig. S6). In the region of 2,250–4,250 μm, passing through the bregma at 2,750 μm, the normalized deformation and area ratio (Fig. 3A) remain unchanged, while mean curvature (Fig. 3B) showed a slight decreasing trend. In this region, the areas of both the isocortex and interbrain exhibited an increasing tendency (Fig. S6), where the interbrain component included the hypothalamus starting at 2,250 μm and the thalamus at 3,250 μm. Our results suggested that residual solid stresses are not uniformly distributed throughout the brain but rather accumulate in specific regions, a phenomenon not observed in prior studies. Understanding the origins and consequences of spatial heterogeneities of solid stresses may provide novel links between the brain structure and function. We normalized all results by the area of the tissue slice to eliminate variations in slices from the anterior to cortical sections, ensuring the reliability of these measurements.

Another factor that is potentially responsible for the changes in solid stress is ECM content. Each region of the brain has specific ECM components that contribute to structural and functional properties and play a significant role in its development. Prior research has demonstrated that ECM proteins, particularly aggrecan, brevican, and tenascin-R, expressed predominantly in mature brains, exhibit a heterogeneous distribution throughout the brain (50). This intricate distribution of ECM components holds significance in brain physiology and may influence solid stress distribution. Furthermore, variations in mechanical properties across different brain regions have been documented; for instance, the corona radiata is stiffer than the basal ganglia in shear but softer in tension, exerting a profound impact on brain mechanical properties and functions (51).

### Residual solid stress in the murine brain depends on ionic strength

**Fig. 4.**
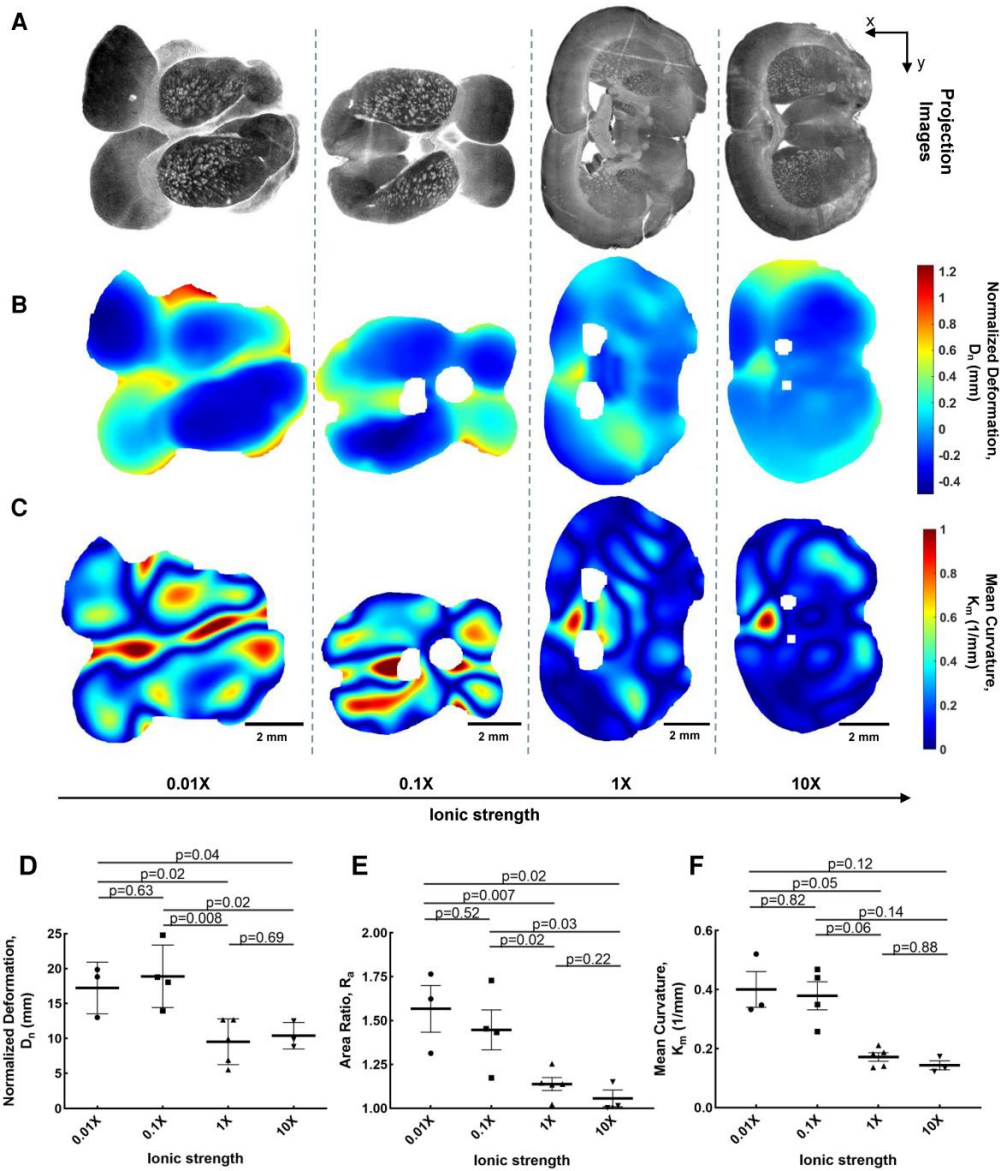
The effect of ionic strength on residual solid stresses. A) Projected microscopy images, B) normalized deformation maps, and C) mean curvature maps of representative brain slices from 8–12 weeks mice under different ionic strength. D) The normalized deformation, E) area ratio, and F) mean curvature show a decreasing trend with increasing ionic strength (mean ± SEM, *n* = 3–5 slices, two-tailed t test).

There is an abundance of negatively charged ECM constituents in brain such as hyaluronic acid, which can contribute to maintenance of solid stresses as in tumors (52) and connective tissues (45, 53). These negatively charged glycosaminoglycans (GAGs) repel each other and induce a local electroosmotic expansion, which can contribute to the observed high level of solid stresses in the brain. We hypothesized that when the ionic strength is decreased, e.g. by decreasing the salt concentration, the charged constituents are further unshielded which increases the effect of expansion forces that ultimately increases solid stress. We measured the solid stress in 8–12 weeks old mice, in different ionic strength of 0.01×, 0.1×, 1×, and 10× PBS. Representative confocal microscopy images, deformation maps, and curvature maps from four tissue slices taken from a similar region of the brain are subjected to different salt concentrations (Fig. 4A–C). At low ionic strength of 0.01× PBS, the brain slices from the similar region of the brain showed larger deformation and larger curvature indicating substantially higher levels of solid stresses. We observed that all the solid stress indices were substantially increased by decreasing the ionic strength (Fig. 4D–F). Unexpectedly, we did not see any significant reduction in normalized deformation, area ratio, and mean curvature when the ionic strength was increased from 1× to 10×. This key observation implies that the electroosmosis associated with the charged constituents of the brain (e.g. GAGs) may contribute to the high level of solid stresses. These components play important roles in tissue morphogenesis through modulating interactions between cell and matrix, cell adhesiveness, and can also binding and affecting growth and differentiation factors, especially the neuronal outgrowth in brain development and regeneration after injury (54). This observation has important implications in diseases where the GAG content in brain is altered, such as Alzheimer's disease, schizophrenia, Parkinson's disease, epilepsy, mucopolysaccharidosis disorders, and brain tumors (54–57). It is noteworthy that the charged components are influenced by the ionic strength in the environment, and concurrently, cells can experience changes in volume due to osmosis, leading to either shrinking or swelling. Future work includes using noncharged solutes such as dextran to perturb osmotic potential without affecting electric charge and differentiating between these two effects on residual solid stress.

### Hemorrhagic stroke results in decreased residual solid stress in murine brain

**Fig. 5.**
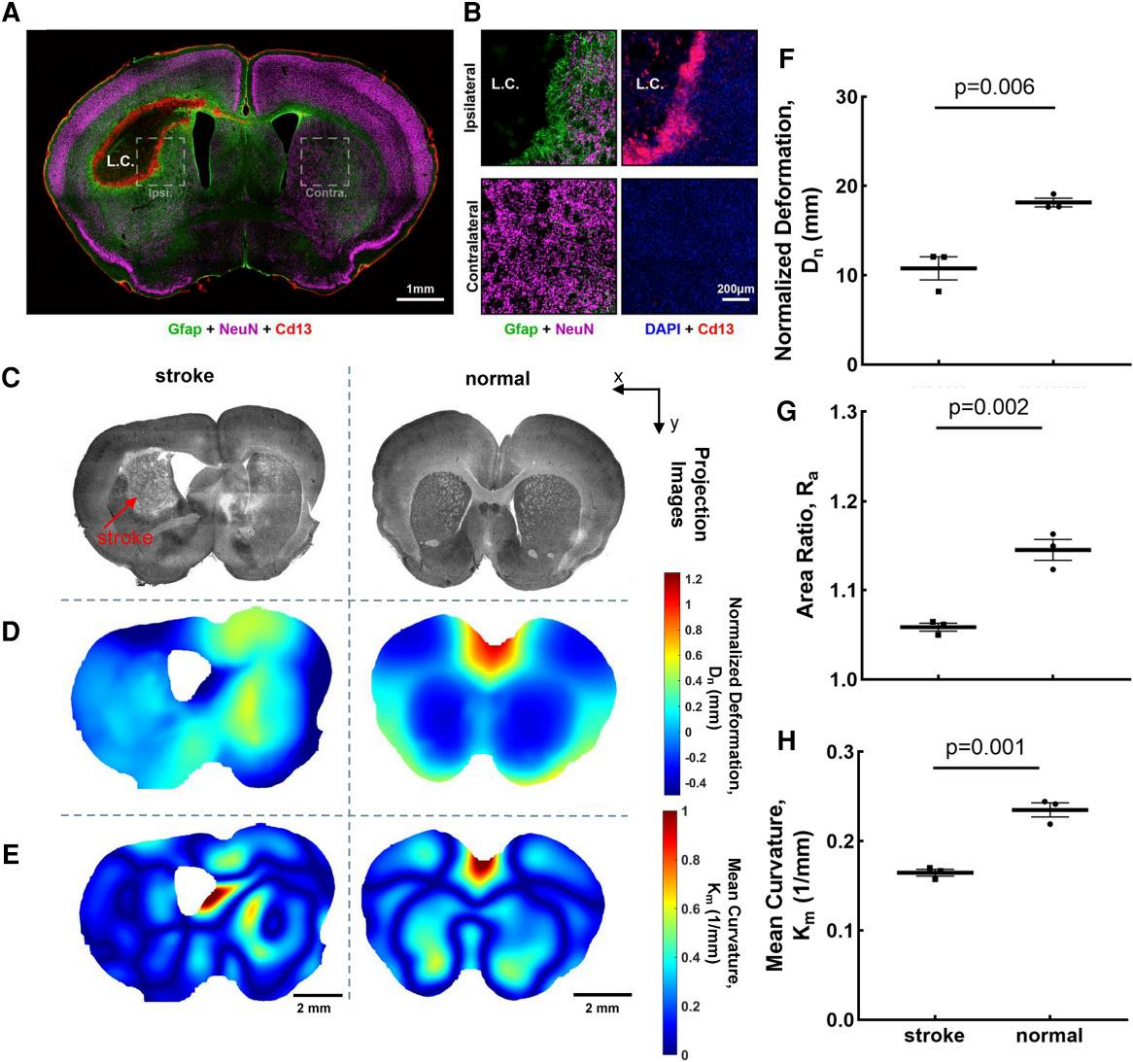
Hemorrhagic stroke results in lower residual solid stress in murine brains. A) The loss of cellular density within the lesion core and the depletion of NeuN-positive neurons on the periphery of the lesion core. B) The infiltrated Cd13-positive immune cells phagocytose cellular debris and in conjunction with locally recruited Gfap-positive astrocytes take part in the formation of the astroglial border. C) The projected image from microscopy, D) deformation maps, and E) mean curvature maps of representative brain sections from hemorrhagic stroke and healthy mice. F) The normalized deformation, G) area ratio, and H) mean curvature are lower in lobes with hemorrhagic stroke compared to normal brain sections (mean ± SEM, *n* = 3 mice, two-tailed t test). Healthy brain sections have higher normalized deformation, area ratio, and mean curvature than hemorrhagic stroke brain sections.

Finally, we decided to probe how a central nervous system (CNS) injury such as hemorrhagic stroke affects the solid stress levels in brain. Hemorrhagic stroke causes a massive and irreversible change in ECM composition and morphology. Initial disruption of local vessels results in the efflux of blood from the damaged vessels, formation of a hematoma and subsequent edema along with the creation of a cytotoxic microenvironment leading to the death of local neural cells (58–60). The death of local neural cells results in the infiltration of monocytes, such as neutrophils, and the subsequent recruitment of peripheral macrophages (61). The infiltrated Cd13-positive immune cells phagocytose cellular debris and in conjunction with locally recruited Gfap-positive astrocytes take part in the formation of the astroglial border, which effectively walls off the region of tissue damage from the preserved, viable CNS tissue (Fig. 5A and B). In addition to the loss of cellular density within the lesion core, there is also a depletion of NeuN-positive neurons on the periphery of the lesion core (Fig. 5A). Due to these structural and cellular alteration in hemorrhagic stroke, we hypothesized that solid stress levels will substantially alter compared to the normal brain. Analysis of the hemorrhagic stroke using the slicing method indeed revealed a loss of symmetry within the brain, as evidenced by gross morphology (Fig. 5C), as well as deformation (Fig. 5D) and curvature (Fig. 5E) mapping of coronal brain sections. Further quantification of the normalized deformation, area ratio, and mean curvature revealed that brains with hemorrhagic strokes had a significant decrease in all three metrics (Fig. 5F–H), demonstrating that hemorrhagic stroke significantly reduced residual solid stress in murine brains. This observation demonstrates that solid stress can be used as a mechanical biomarker which is sensitive to cellular and structural changes in the brain. Furthermore, the reduction of solid stresses in the hemorrhagic stroke lesion may contribute to the pathophysiology of stroke due to mechanoresponsiveness of immune and brain cells. Such mechanical cues may lead to identification of new biomarkers and therapeutic targets in future studies. It has been observed that CNS tissue exhibits a softening effect after injury, a deviation from the typical response observed in other common tissues where scar formation generally results in increased stiffness compared to the surrounding healthy tissue (22). There also existed an increased expression of ECM components, including vimentin, glial fibrillary acidic protein (GFAP), laminin, and collagen IV, at the injured tissue sections (22), which contribute to tissue elasticity and present a promising avenue for future research to delve into their potential correlation with the reduction of residual solid stress in brain hemorrhagic stroke.

## Discussion

In this study, we quantified and mapped residual solid stress in murine brains at three different stages during the mouse lifespan at 5–7 days, 8–12 weeks, and 22 months. We utilized our previously developed tissue slicing method (3, 35) to quantify the deformation induced by relaxation of the intrinsic solid stress. We quantified three separate indices of solid stress—normalized deformation, area ratio, and mean curvature—and validated our method against both a positive control and negative control. Our study revealed a significant escalation in residual solid stress within the murine brain from infancy to adulthood, persisting at heightened levels throughout aging. This observed trend could be linked to alterations in brain components, particularly the heterogeneities of gray matter and white matter (26). Furthermore, our exploration unveiled age-dependent variations in the viscoelastic properties of the murine brain, likely stemming from the distinct viscoelasticity of brain constituents and their age-related changes (44). We created the first map of solid stress through serial coronal sections up to 5 mm into the brain. Notably, we observed that posterior sections of the mature murine brain exhibited higher solid stresses compared to anterior sections. This disparity may be attributed to the heterogeneously distributed ECM components and region-specific mechanical properties (50, 51). Additionally, we identified that solutions with lower ionic strength amplify solid stress levels in the murine brain. However, further investigation is required to disentangle the effects of charged ECM components (e.g. GAGs) and changes in cell volume due to osmosis. Finally, our study demonstrated a reduction in solid stress in the case of a hemorrhagic stroke in the mature murine brain. This decrease could potentially be linked to alterations in ECM composition and morphology (58–60). The insights garnered from this study contribute to a better understanding of solid stress as a potential biomarker for aging and various brain pathologies.

One of the limitations of this study is the invasiveness of our approach to measure solid stress, which method cannot be used in human brains in vivo. To demonstrate solid stress as a sensitive biomarker, a noninvasive method is required to probe the longitudinal changes in solid stress in healthy and diseased brains. Potential solutions are the recently developed methods where a sensor of solid stress, e.g. deformable microgels, is embedded in the organ of interest (18, 36–39). While these methods are either minimally invasive during the injection of the sensor (39), the sensor can potentially be embedded during the early development as in the case of tumor growth (18). Developing a method to noninvasively study solid stresses in healthy brain of animals and human remains an unmet need with immense potential for uncovering the role of mechanical forces in brain development and aging.

Another unmet need is the in vitro and in vivo models of solid stress in brain. We previously developed in vivo models to apply chronic compressive stresses on brain (20, 21) to recapitulate the compressive forces applied by tumors onto the surrounding brain tissue. However, a model system that is able to faithfully recapitulate the normal growth-induced solid stresses in brain is still an unmet need. Such in vitro or in vivo models can substantially improve our understanding of mechanobiology and mechanoimmunity associated with solid stress in brain development, aging, and disease.

## Materials and methods

Additional methods are included in the Supplementary material.

### Mice strain

All experiments conformed to ethical principles and guidelines under protocols approved by the Boston University Institutional Animal Care and Use Committee. Mice were housed and bred under pathogen-free conditions at the Boston University Animal Science Center. Mice were C57BL/6 strain from two age groups: 5–7 days, 8–12 weeks, and mice from 22 months age group were C57BL/6J strain. A breeding pair of transgenic B6.129(Cg)-Gt(ROSA)26Sortm4(ACTB-tdTomato,-EGFP)Luo/J (JAX #007676, Jackson Laboratory, ME, USA), hereafter referred to as mTmG, was purchased to start a colony and was the primary source of all animals for the experiments. mTmG mice were used in the 5–7 days and 8–12 weeks age groups and endogenously expressed tdTomato fluorescence in all cell membranes. Mice in the 22 months age group were wild type. *n* = 5 mice were used for all age groups.

### Slicing method to measure residual solid stresses

#### Releasing residual solid stress

We used our previously developed tissue slicing method (3). 2% weight-by-volume agarose is prepared using low gelation temperature agarose (Sigma-Aldrich) mixed with PBS. The 2% agarose solution is in a liquid state at a temperature of 40 °C. The organs, including brain and kidney, were immediately extracted posteuthanasia through CO_2_, and embedded in 2% agarose in a stainless-steel cast provided by the commercial vibrating microtome, Compresstome (F00395A, Precisionary Instruments LLC). The agarose-embedded organs are fully immersed in PBS and sliced via the Compresstome to the desired thickness of 250 μm which is thin enough for showing the deformation, but thick enough to avoid the tissue being broken by handling (Fig. 1A). The tissue slice is detached from the agarose slice spontaneously or manually with a pair of sharp tweezers, collected in a 24-well plate, and immersed in PBS for 20 min at room temperature to allow the tissue to release any residual solid stresses in the tissue (Fig. 1B). As a result of stress relaxation, the tissue slice becomes deformed. Then the PBS is removed, and the slice is embedded in 1% agarose to hold the deformation. The tissue slice is fixed for 4 h in 10% formalin to prevent the tissue degradation, and then was washed and stored in PBS at 4 °C (Fig. 1C). Mice in the 22 months age group were wild type and the tissue slices were stained with DAPI (Thermo Fisher), which is a fluorescent nuclear and chromosome counterstain and can emit blue fluorescence.

#### Imaging the stress-induced deformation

With the fluorescence reporter in the tissue, the sample is imaged via Olympus FV3000 laser scanning confocal microscope using UPL SAPO10X2 (Olympus, NA 0.4, 10× magnification) air immersion objective lens (Olympus) at scanning resolutions between 512 × 512 pixels in FV31S-SW Viewer software (Olympus). Slices from 5–7 days and 8–12 weeks groups mice had tdTomato fluorescence and were imaged using a 561 nm excitation laser and 570–620 nm detection wavelength, the slices from 22 months group mice had DAPI fluorescence and were imaged using a 405 nm excitation laser and 430–470 nm detection wavelength. The tissue-agarose construct that is immersed in PBS was imaged to get the 3D structure (Fig. 1D).

#### Postprocessing of the stress-induced deformation

The 3D images (Fig. 1I) are exported from ImageJ to MATLAB for postprocessing. The postprocessing includes normalized deformation calculation and the reconstruction of the deformation map. The release of residual solid stress via slicing results in surface bending of the slice. The overall bending extent after slicing represents the area strain, a quantitative index for residual solid stress in the slice. We defined the normalized deformation, *D*_n_, as the average height difference from the curved surface to the midline (Fig. 1E):


$$D_{n}=\frac{\sum_{k}^{N}\left|z_{k}-\bar{z}\right|}{N}.$$

Area ratio, *R*_a_, is defined as the ratio of surface area, *A*_s_, to projection area, *A*_p_, represents the deformation extent (Fig. 1F):


$$R_{a}=\frac{A_{s}}{A_{p}}.$$

The deformation map shows the distribution of deformation in individual slices (Fig. 1J). Mean curvature, *K*_m_, is defined as the average of mean curvature on tissue slices and indicates the degree of deformation curved (Fig. 1G). The curvature maps show the distribution of curvature in individual slices (Fig. 1K):


$$K_{m}=\frac{\sum_{k}^{N}|\frac{1}{2}\left(\frac{1}{r_{k1}}+\frac{1}{r_{k2}}\right)|}{N}.$$

### Measurement of viscoelastic properties

#### Unconfined compression test

The bulk viscoelastic properties of the tissues were measured via the Instron 5900 Series System (Illinois Tool Works Inc.) in macroscale with an unconfined compression test. Organs were sliced with the Compresstome using the slicing method with a 2 mm thickness. The slices were then punched out with 6 mm diameter biopsy punches at the center of brain tissue to obtain a cylinder shape and discard the surrounding agarose hydrogel. The punched tissue was maintained in PBS with protease inhibitors at 4 °C before mechanical testing. All measurements were performed in near-physiological PBS at room temperature. The sample was placed in a loading machine and the displacement was zeroed at the point where the upper plate was in contact with the sample, then applying a constant strain. Each specimen was compressed by 5% of original height in ramps of 1 s and allowed to relax for 3 min. Four consecutive steps were performed to apply the displacement changing with time. The Instron then measured the force changes in the whole process, including the increase of force while compressing, and the force decreasing during the relaxation time till it reached an equilibrium point. The plots of displacement vs. time and force vs. time were converted to plots of stress vs. time and strain vs. time (Fig. S5). Stress modulus, *σ*, was the ratio of force to surface area, and strain, *ε*, was the ratio of displacement to total thickness. The stress is then plotted as a function of strain, and Young's modulus, *E*, is estimated as the slope of the linear fit to the stress–strain data as modulus measurement (Fig. S5). We observed a reasonable linear relationship between stress and strain within 20% strain, and to keep the model simple with minimum number of parameters, we chose a linear model to report Young's modulus. However, for higher strains, nonlinear hyperelastic models might be more appropriate:


σ=Eε+C.

Instantaneous/equilibrium modulus ratio, *R*_s_, is the ratio of maximum modulus, *σ*_max_, to equilibrium modulus, *σ*_equilibrium_, which indicates how much stress the tissue releases to reach an equilibrium point (Fig. S5):


$$R_{s}=\frac{\sigma_{\max}}{\sigma_{\mathrm{equilibrium}}}.$$

The relaxation time constant, τ, was used to compare the time of how long it takes for the tissue stress level to relax (Fig. S5), and can be divided into fast relaxation time constant, τ_fast_ and slow relaxation time constant, τ_slow_, for accurate evaluation:


$$\sigma (t)=a_{1}e^{-\frac{t}{\tau_{\mathrm{fast}}}}+a_{2}e^{-\frac{t}{\tau_{\mathrm{slow}}}}+b$$

### Statistical analysis

The data are presented as mean ± SEM. Statistics between every two groups of data are calculated as two-tailed t test (*n* ≥ 3) to determine significance or *P*-values. Statistics among multiple datasets are calculated as one-way ANOVA test to determine significance or *P*-values. All *P*-values are reported in figures to observe statistical trends of data.

## Acknowledgment

We thank the support of Boston University Micro and Nano Imaging Facility under award no. S10OD024993. The 22 months mice were provided from Dr Brianne Connizzo's lab, which were initially obtained from NIH/NIA Aged Rodent Colony. The content is solely the responsibility of the authors and does not necessarily represent the official views of the National Institutes of Health.

## Supplementary Material

pgae141_Supplementary_Data

## Data Availability

All data are available in the main text or the Supplementary material. Raw data and code for analysis are deposited in a Zenodo repository and is available from (https://doi.org/10.5281/zenodo.10737544).

## Preprints

This manuscript was posted on a preprint: https://doi.org/10.1101/2023.09.25.559368.
