# Supplementary material for: Alteration of mechanical stresses in the murine brain by age and hemorrhagic stroke
Source: PNAS Nexus. 2024 Apr 24;3(4):pgae141. doi: 10.1093/pnasnexus/pgae141 (PMC11042661; doi:10.1093/pnasnexus/pgae141)
Supplement: pgae141_Supplementary_Data [file pgae141_supplementary_data.pdf]

Alteration of mechanical stresses in the murine brain by age and hemorrhagic stroke

Siyi Zheng[1], Rohin Banerji[1], Rob LeBourdais[1], Sue Zhang[1], Eric DuBois[1], Timothy O'Shea[1], Hadi T. Nia[1]*

[1]Department of Biomedical Engineering, Boston University, Boston, MA 02215

Corresponding author:

Hadi T. Nia (htnia@bu.edu)

Room 206, 36 Cummington Mall

Boston, MA 02215

Phone: 617-353-2805

Author Contributions: S.Zheng and H.T.N designed the study; S.Zheng, S.Zhang, and E.D performed research; S.Zheng, R.B, R.L, and H.T.N contributed to data analysis; S.Zheng analyzed data; S.Zheng, R.B, R.L, S.Zhang, E.D, T. O., and H.T.N wrote the manuscript.

Competing Interest Statement: Authors declare that they have no competing interests.

Data and materials availability: All data are available in the main text or the supplementary materials. Raw data and code for analysis are deposited in a Zenodo repository and is available from (doi.org/10.5281/zenodo.10737544).

Classification: Biological, Health, and Medical Sciences/Biophysics and Computational Biology; Physical Sciences and Engineering/Bioengineering

Keywords: Residual solid stress, mouse brain, age-related alteration, hemorrhagic stroke

This PDF file includes:

- Supplementary notes
- Supplementary methods
- Supplementary figures with captions

Supporting Information

Supporting Information Text

The supporting information includes additional methods which are used to test the validation of our slicing method and evaluate the accuracy of measurement and quantification. Additionally, we also evaluate the potential relationship between residual solid stress changes in the mouse brain and the volume changes in the human brain.

Methods

Slicing method to measure residual solid stresses

Evaluating the effect of Ionic strength on residual solid stresses

In the slicing method, the slices are immersed in PBS for deformation due to stress relaxation after the fresh tissue been sliced. In tissue experiments, PBS is often used to maintain tissue hydration during mechanical testing, the solutes from the buffer can diffuse into the tissue and interact with its structure and mechanics (1). As the ionic strength is understood to have effect on tissue mechanics (2), the relationship between the ionic strength in PBS and the deformation on the slices is studied. Four groups of ionic strength are set through using different concentration of PBS, including 0.01X, 0.1X, 1X, and 10X PBS. Among the four groups, the 1X PBS is the physiological condition and is used in normal experiment and acts as the control group at here. The slices are immersed in specific buffer immediately after slicing for tissue deformation. Then hold the deformed slices in 1% agarose made by corresponding 0.01X, 0.1X, 1X and 10X PBS, fixed with formalin and imaged with confocal microscopy, following by tissue imaging and post-processing.

Evaluating the spatial distribution of residual solid stress distribution in whole brain

The whole brain from mice aged 8–12 weeks was sliced to get 18 continuous coronal sections with a thickness of 250 μm each, for a total of 4500 μm. Then builds deformation and curvature maps, and quantification of normalized deformation, area ratio, and mean curvature to study the distribution of residual solid stress in brain at the coronal direction.

Induction of Hemorrhagic Strokes

All surgical procedures were approved by the BU IACUC (Protocol number: PROTO20200045) and conducted within a designated surgical facility. All procedures were performed on C57BL/6 mTmG mice that were aged 8-12 weeks at the time of surgery under general anesthesia achieved through inhalation of isoflurane in oxygen-enriched air. Collagenase was used to induce hemorrhagic strokes. Shaved mice heads were stabilized and horizontally leveled in a stereotaxic apparatus using ear bars (David Kopf, Tujunga, CA). A small craniotomy over the left coronal suture was performed using a high-speed surgical drill and visually aided by an operating microscope. A small rectangular flap of bone encompassing sections of the frontal and parietal bone was removed to expose the brain in preparation for injection. To induce hemorrhagic strokes, 1 μL of Collagenase I (Gibco™ 17018029) (0.1 U/μL in sterile PBS) was injected into the caudate putamen nucleus at 0.15 μL/min using target coordinates relative to Bregma: +1.0 mm A/P, +2.5 mm L/M and −3.0 mm D/V. A standard micropipette injection protocol was used to make all injections into the brain using pulled borosilicate glass micropipettes (WPI, Sarasota, FL, #1B100-4) that were ground to a 35° beveled tip with 150–250 μm inner diameter. Glass micropipettes were mounted to the stereotaxic frame via specialized connectors and attached, via high-pressure polyetheretherketone (PEEK) tubing, to a 10 μL syringe (Hamilton, Reno, NV, #801 RN) controlled by an automated syringe pump (Pump 11 Elite, Harvard Apparatus, Holliston, MA).

Histology

Transcardial perfusions for Immunohistochemistry: After terminal anesthesia by overdose of isoflurane, mice were perfused transcardially with heparinized saline (10 units/ml of heparin) and 4% paraformaldehyde (PFA) that was prepared from 32% PFA Aqueous Solution (Cat# 15714, EMS), using a peristaltic pump at a rate of 7 mL/min. Approximately, 10 mL of heparinized saline and 50 mL of 4% PFA was used per animal. Brains were immediately dissected after perfusion and post-fixed in 4% PFA for 6-8 hours. After PFA post-fixing, brains were cryoprotected in 30% sucrose + 0.01% sodium azide in Tris-Buffered Saline (TBS) for at least 3 days with the sucrose solution replaced once after 2 days and stored at 4°C until further use.

Immunohistochemistry: Coronal brain sections (40 µm thick) were cut using a cryostat (Microm, HM 525). Tissue sections were stored in TBS buffer + 0.01% sodium azide at 4°C. Tissue sections were processed for immunofluorescence using free floating staining protocols described in comprehensive detail previously using donkey serum to block and triton X-100 to permeabilize tissue (3-5). The primary antibodies used are rat anti-Gfap (1:1000, Thermofisher, #13-0300); goat anti-Cd13 (1:500, R&D Systems, AF2335); guinea pig anti-NeuN (1:500, Synaptic Systems, #266 004). All secondary antibodies purchased from Jackson ImmunoResearch Laboratories, with donkey host and target specified by the primary antibody. Secondary antibodies were diluted 1:250 prior to incubation. Cell nuclei were stained with 4',6'-diamidino-2-phenylindole dihydrochloride (DAPI; 2 ng/ml; Molecular Probes). Stained sections were imaged using epifluorescence and deconvolution epifluorescence microscopy on an Olympus IX83. Subsequently, images were prepared for publication using NIH Image J (1.53) software.

Positive and negative control organs

The heart is studied previously as an organ which has a strong residual stress in it (6). And can be used to test the deformation quantification accuracy of showing the extent of residual solid stress. The same slicing method is used to slice the mouse heart, compared the deformation and curvature maps and quantification of normalized deformation, area ratio, and mean curvature between kidney and heart.

Evaluating the buoyancy effect

Since the tissue slice was immersed in PBS for the deformation occur, flipping test is done to avoid the possibility that the deformation was an artifact caused by the buoyancy. The buoyancy might have different pressure acting on opposite sides of the slice, and thus the heavy part will sink and the light part will go up and floating in the liquid, and result to deformation. Imaging the slice immediately after tissue deformed, and then flip the slice to upside down and then image again. These all steps will be finished before the tissue degradation. Then compared the cross-section view and the quantification of normalized deformation, area ratio, and mean curvature.

Evaluating the effect of fixation

In the slicing method, formalin is used to fix the tissue and avoid tissue degradation, the potential artifact of fixation needs to be evaluated. The brain slice is imaged after it deformed and hold in 1% agarose, and then do the fixation with formalin, wash with PBS, after that did another imaging, and compare the cross-section view, deformation and curvature maps, and quantification of normalized deformation, area ratio, and mean curvature.

Fitting the changing tendency between residual solid stress and brain volume

The mouse age is converted to human age as showed in previous study (7), that 5–7 day mice are equivalent to 0.1 human years, 8-12 week mice are equivalent to 20 human years, and 22 month mice are equivalent to 80 human years. The changing tendencies of normalized deformation, area ratio, and mean curvature in mouse brain with respect to age closely trend with changes in brain volume as humans age (8-10).

Supplementary References

1. B. N. Safa, K. D. Meadows, S. E. Szczesny, D. M. Elliott, Exposure to buffer solution alters tendon hydration and mechanics. *J Biomech* **61**, 18-25 (2017).
2. A. M. Nguyen, M. E. Levenston, Comparison of osmotic swelling influences on meniscal fibrocartilage and articular cartilage tissue mechanics in compression and shear. *J Orthop Res* **30**, 95-102 (2012).
3. T. M. O'Shea *et al.*, Foreign body responses in mouse central nervous system mimic natural wound responses and alter biomaterial functions. *Nat Commun* **11**, 6203 (2020).
4. M. A. Anderson *et al.*, Required growth facilitators propel axon regeneration across complete spinal cord injury. *Nature* **561**, 396-400 (2018).
5. T. M. O'Shea *et al.*, Lesion environments direct transplanted neural progenitors towards a wound repair astroglial phenotype in mice. *Nat Commun* **13**, 5702 (2022).
6. L. A. Taber, Biomechanics of cardiovascular development. *Annu Rev Biomed Eng* **3**, 1-25 (2001).
7. S. Dutta, P. Sengupta, Men and mice: Relating their ages. *Life Sci* **152**, 244-248 (2016).
8. M. Kozlov, Your brain expands and shrinks over time - these charts show how. *Nature* **604**, 230-231 (2022).
9. R. A. I. Bethlehem *et al.*, Brain charts for the human lifespan. *Nature* **604**, 525-533 (2022).
10. S. Marek *et al.*, Reproducible brain-wide association studies require thousands of individuals. *Nature* **603**, 654-660 (2022).
11. A. R. Atlas, Mouse Brain [brain atlas]. Available from atlas.brain-map.org. atlas.brain-map.org.
12. E. S. Lein *et al.*, Genome-wide atlas of gene expression in the adult mouse brain. *Nature* **445**, 168-176 (2007).
13. J. A. Harris *et al.*, Hierarchical organization of cortical and thalamic connectivity. *Nature* **575**, 195-202 (2019).
14. S. W. Oh *et al.*, A mesoscale connectome of the mouse brain. *Nature* **508**, 207-214 (2014).

Supplementary Figures

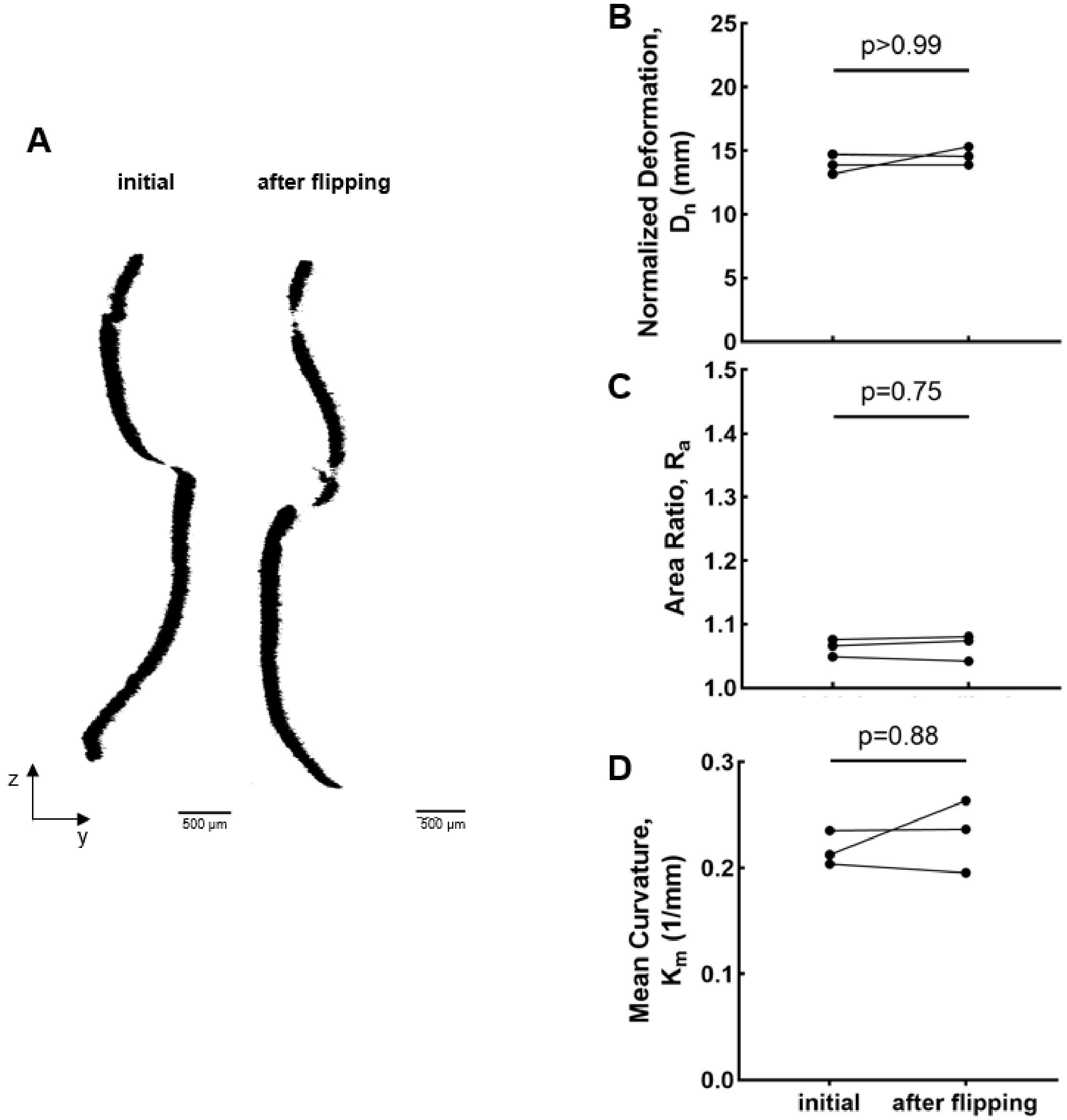

Fig. S1. Buoyancy does not affect residual solid stress quantification. (**A**) The orthogonal view of representative microscopy images of tissue slices before and after flipping. (**B**) Statistics of normalized deformation, (**C**) area ratio, and (**D**) mean curvature between slices initial and slices after flipping (mean ± SEM, n=3 slices, two-tailed t-test). All normalized deformation, area ratio, and mean curvature have no significant difference of brain slices between initial and after flipping stages.

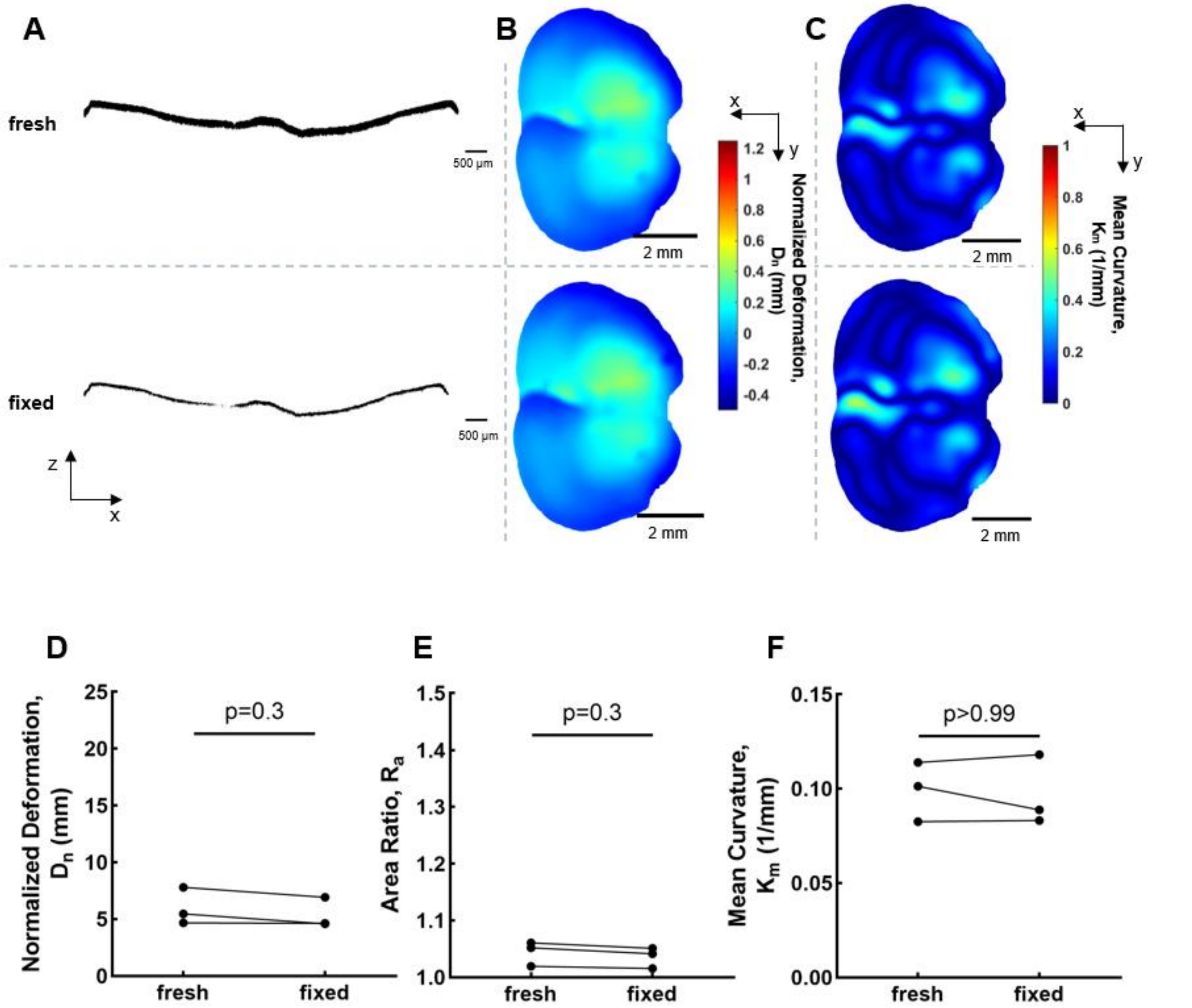

Fig. S2. Fixation does not affect residual solid stress quantification. (**A**) The orthogonal view of microscopy images of the tissue slices, (**B**) deformation maps, and (**C**) mean curvature maps of representative brain slices from fresh and fixed states. Statistics of (**D**) normalized deformation, (**E**) area ratio, and (**F**) mean curvature between fresh and fixed brain slices (mean ± SEM, n=3 slices, two-tailed t-test). All normalized deformation, area ratio, and mean curvature have no significant difference of brain slices between fresh and fixed stages.

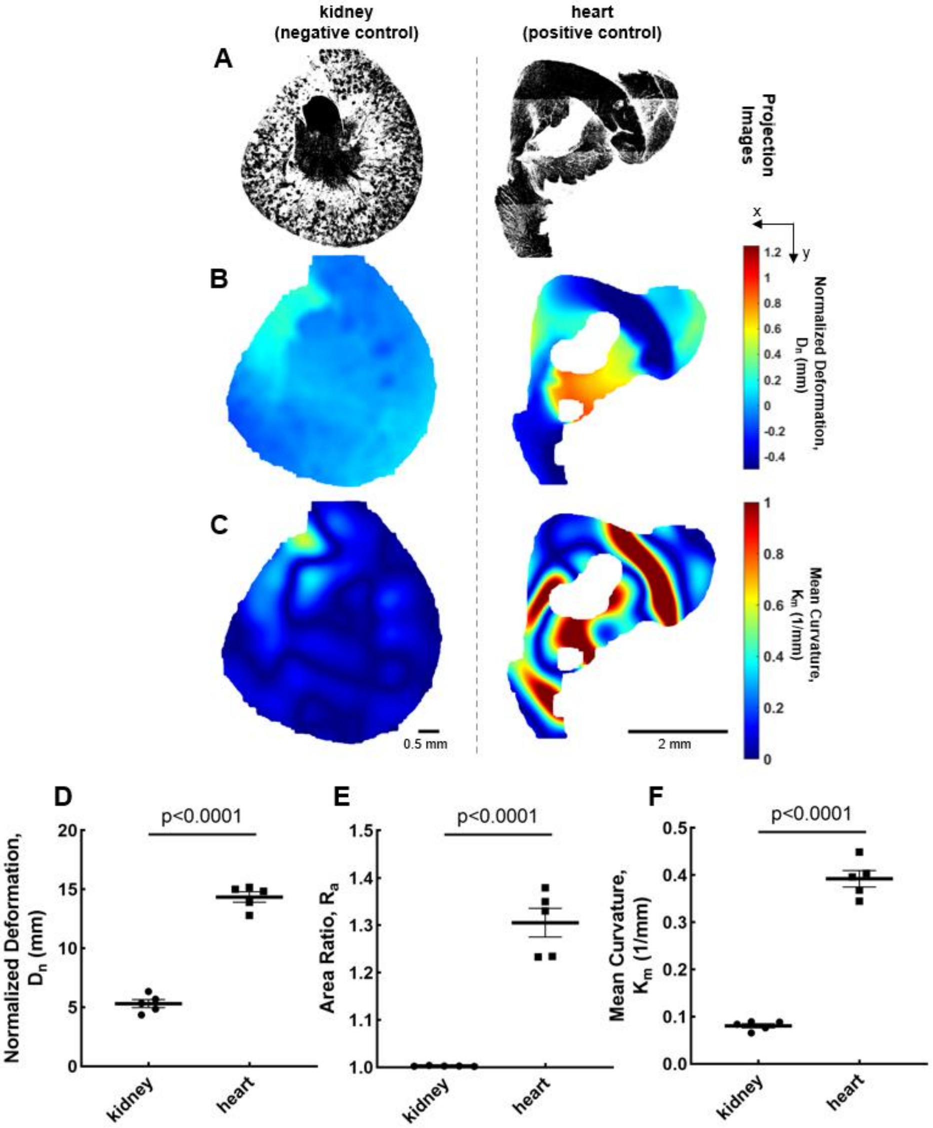

Fig. S3. Higher residual solid stress exists in heart. (**A**) Projected microscopy images, (**B**) corresponding deformation maps, and (**C**) mean curvature maps of representative slices from kidney and heart. Statistics of (**D**) normalized deformation, (**E**) area ratio, and (**F**) mean curvature among kidney and heart slices (mean ± SEM, N=5 mice, two-tailed t-test).

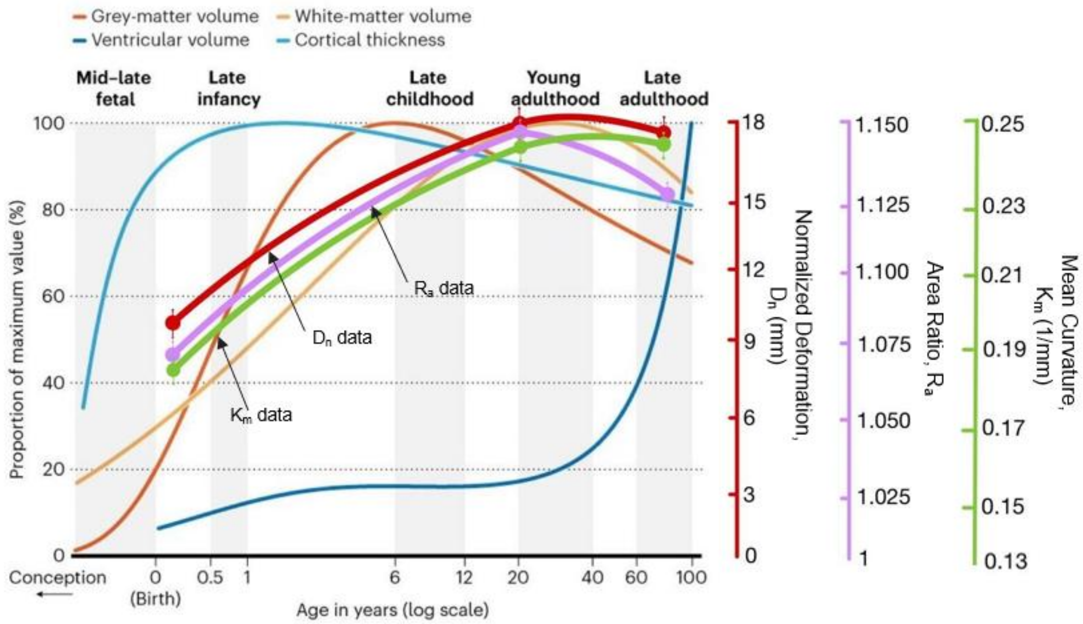

Fig. S4. The comparison between volume change in human brain and residual solid stress change in mouse brain. Convert mouse lifespan to human (7) where 5–7 day, 8-12 week, and 22 month mice are equivalent to 0.1, 20, and 80 years in human, respectively. The normalized deformation, D_n, area ratio, R_a, and mean curvature, K_m, trend with age in the brain compared to the volume changes. Modified from (8-10).

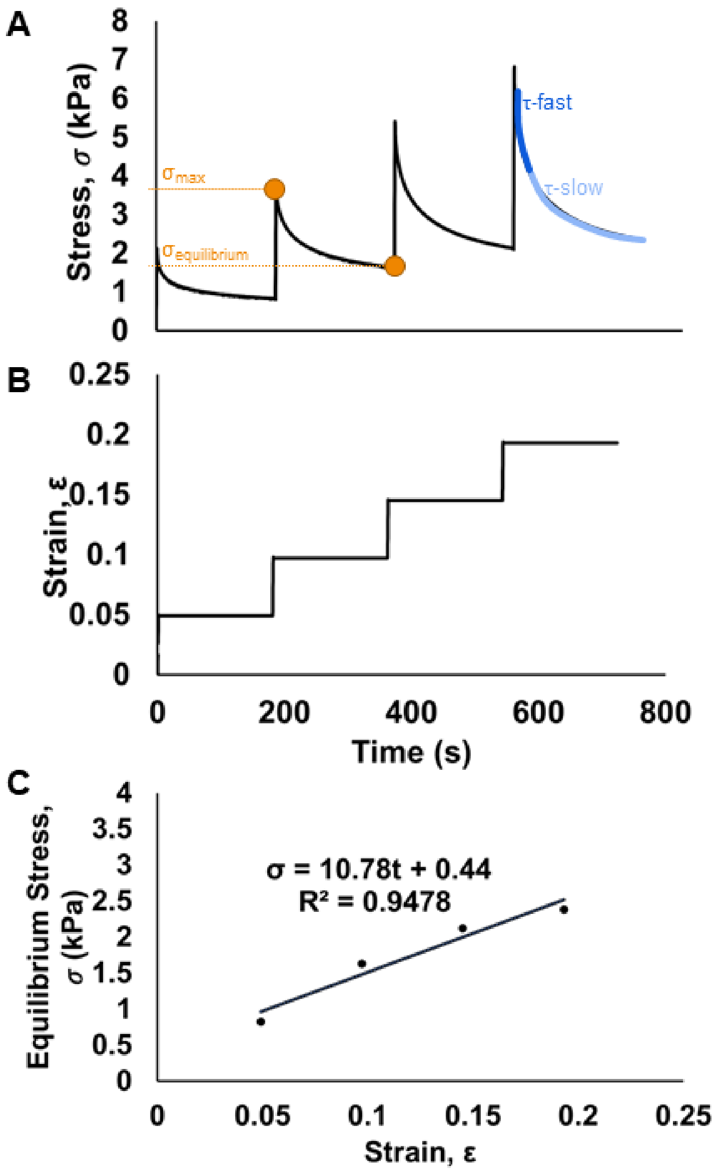

Fig. S5. Methodology for quantification of viscoelastic properties. (A) The stress relaxation is measured with an unconfined compression test on an Instron. From the curves, the instantaneous / equilibrium modulus ratio, R_s, is the ratio of maximum, σ_{max}, and equilibrium modulus, $\sigma_{equilibrium}$ in each step, indicating how much stress the tissue released to reach a lower equilibrium point. Relaxation time constant, τ, evaluates the time of how long it takes for the tissue stress level to relax and become stable, and can be divided into fast, τ_{fast}, and slow parts, τ_{slow}. (**B**) Four steps each of 5% compressive is applied and held for 3 minutes to let the stress relax. (**C**) The equilibrium stress is plotted as a function of strain and the Young's modulus is estimated as the slope of the linear fit to the stress-strain data.

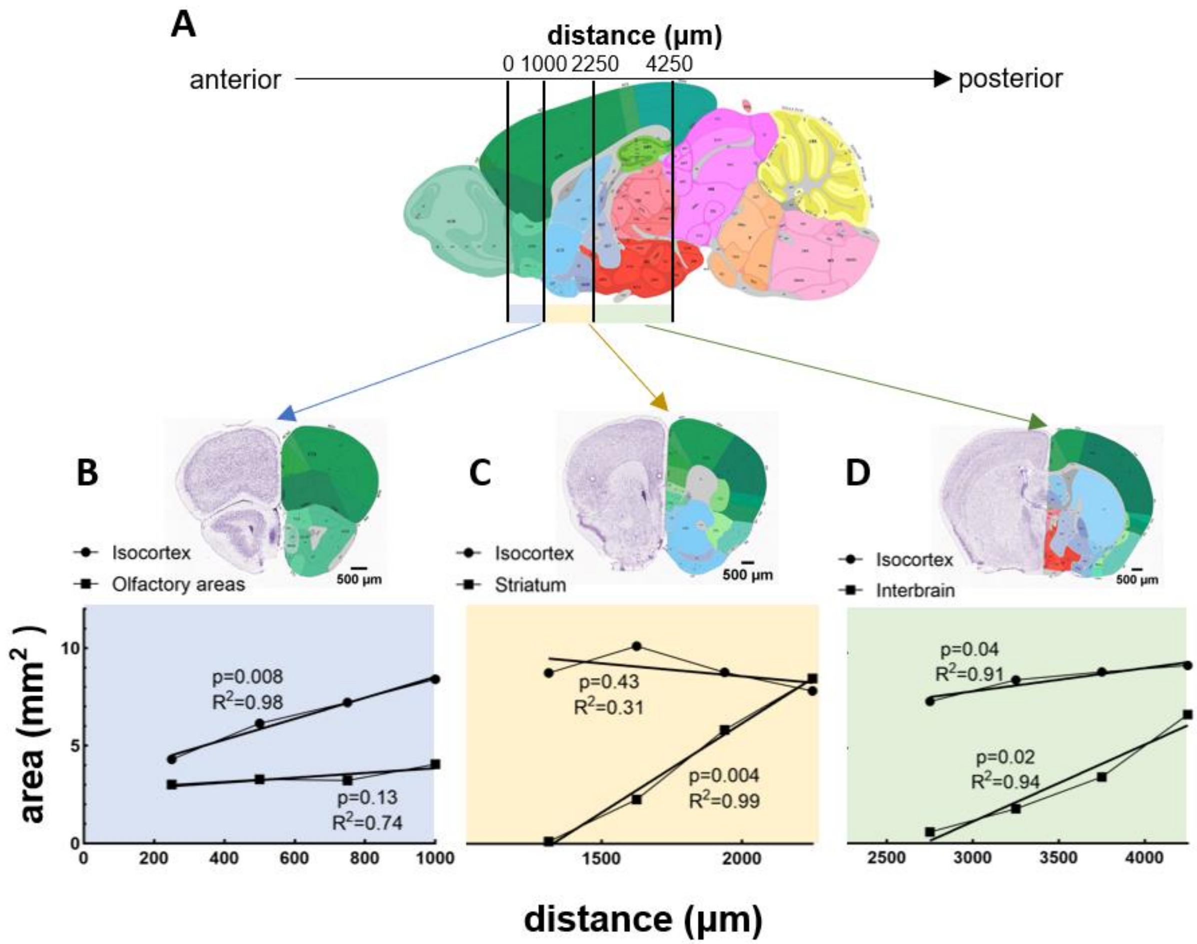

Fig. S6. The areal changes of brain components. (**A**) Based on the brain regions that we sliced for solid stress measurement, we divided the brain into three region and compared the areal changes of different anatomical regions in each compartment based on existing brain atlases (11-14). Area was computed from the labeled regions of the hemisphere in the brain atlas. (**B**) Area changes of isocortex and olfactory areas from 0-1000 μm. (**C**) Area changes of isocortex and striatum from 1000-2250 μm (**D**) Area changes of isocortex and interbrain from 2250-4250 μm. A representative image was selected from the published brain atlas (11) for each defined region.
